# Performance Summary Display Ontology: Feedback intervention content, delivery, and interpreted information

**Published:** 2022-09

**Authors:** Zach Landis-Lewis, Cooper Stansbury, John Rincón, Colin Gross

**Affiliations:** 1 University of Michigan Medical School, Ann Arbor, Michigan, USA; 2 Harvard Medical School, Cambridge, Massachusetts, USA

**Keywords:** feedback intervention, audit and feedback, visualization, quality improvement, behavior change

## Abstract

Feedback loops are vital for decision-making and behavior change in health systems, but not all feedback is of equal value. Clinical performance feedback to healthcare professionals and teams has potential for large effects on clinical practice, but evidence suggests that low-value performance feedback is widespread. A primary barrier to understanding the value of feedback loops in health systems may be a lack of a well-defined model and shared semantics for the information that they carry. An ontology for audit and feedback research may be used to address these issues by standardizing feedback intervention metadata. Research describing feedback interventions recognizes differences between the content of the feedback and its delivery process. However, terms describing feedback intervention content are inconsistent, and appear to vary considerably between audit and feedback frameworks, which can result in confusion around what is being delivered in a performance summary. Our objective was to develop an ontology of a performance summary in a clinical performance feedback intervention for the purposes of standardizing metadata. We developed the Performance Summary Display Ontology (PSDO) iteratively by 1) identifying terms for classes from behavior change theories relating to feedback interventions and cognitive theories of visualization, 2) searching for relevant existing ontologies and classes, and 3) using the terms to specify information content and visual displays in published examples of dashboard displays and feedback reports. PSDO is a lightweight application ontology that specifies performance information content and its representations for the purpose of feedback intervention research and evaluation. PSDO contains 3 primary domains: 1) Performance information content, based on constructs from behavior change theories, 2) Marks and their qualities, based on constructs from visualization theories, and 3) roles that link marks, information content, and other emergent characteristics, as interpreted information. PSDO may enable standardization of metadata for the study of feedback interventions.

## 1. Introduction

Feedback loops are vital for decision-making and behavior change in health systems, but not all feedback is of equal value. Clinical performance feedback to healthcare professionals and teams, also known as *audit and feedback*, has potential for large effects on clinical practice[1]. Unfortunately, decades of evidence from hundreds of trials demonstrates persistent mixed effects[2,3], suggesting that low-value performance feedback is widespread. The need to better understand the value of feedback gains importance as healthcare organizations expand digital infrastructure for performance feedback in the form of dashboards and email communication for population health and quality improvement[4–6].

A primary barrier to understanding the value of feedback loops in health systems may be a lack of a well-defined model and shared semantics for the information that they carry. In the audit and feedback research community, models of feedback are developed from varied disciplines, including psychology, sociology, and informatics[7–12]. The resulting set of foundational theoretical constructs contribute various terms and definitions for the *content* of feedback. For example, differing terms are used to refer to feedback content about social comparison, including *benchmarks*[8], *normative information*[12], and *others’ previous performance*[7]. The same terms are used with differing definitions, leading to confusion, such as *trend*, which has been alternately defined as a comparison to one’s past performance[8,9] and a change in performance over time[13], and *velocity*, defined as feedback intervention frequency[14] and the amount of change in performance since the previous feedback intervention[12].

An important advance to standardize the description of these elements and processes is the Clinical Performance Feedback Intervention Theory, (CP-FIT), which incorporates constructs from more than 30 behavior change theories into a single theory, using evidence synthesized from qualitative studies of feedback interventions in healthcare[9]. CP-FIT is a valuable theory that can be used to interpret evidence about audit and feedback, and to guide future research. To our knowledge, CP-FIT lacks ontologically consistent definitions, resulting in potential semantic ambiguity that prevents the effective organization of research data and evidence synthesis[15]. An ontology for audit and feedback research may be used to address these issues by standardizing feedback intervention metadata, and for the refinement of CP-FIT and other theories contributing to our knowledge of the value of feedback in healthcare.

## 2. Background

### 2.1. Feedback content vs delivery

*Audit and feedback* is widely understood as a process of delivering a summary of clinical performance to healthcare professionals and teams[1,16]. Thus, *audit and feedback* has an *audit* component, in which a *performance summary* is developed, followed by a *feedback* component, in which the performance summary is delivered. Given its central role in audit and feedback, the performance summary may be an important starting place for ontology development.

Research describing feedback interventions recognizes differences between the *content* of the feedback and its *delivery process*[7,9,12]. However, terms describing *feedback intervention content* are inconsistent, and appear to vary considerably across audit and feedback frameworks (Table 1). This ambiguity can result in confusion around what is being delivered in a performance summary.

Differentiating the *information content* from its process of delivery is also an issue for visualizations, which are described inconsistently as part of the delivery process of the performance summary (e.g. *feedback delivered graphically*[12]) and as part of the content of the performance summary (*graph presented*[7], *graphical elements*[9]). Visualization theories have a potentially significant role to play in the modeling of feedback content and delivery aspects, due to the potential for visualizations to strongly influence performance information interpretation[17] and to negatively moderate the effect of feedback interventions[12].

Visualization theories offer constructs to clarify relationships between the physical marks (made of ink or pixels) and perceived entities such as areas, points, and lines in a visualization[18]. A cognitive theory of visualizations called *relational information displays* further specifies relationships between information content and visualizations, by recognizing alignment between the physical characteristics of marks (e.g. length, slope) and the properties of entities that they represent (e.g. performance, rate of change) [19,20]. Visualization theories also offer terms and relationships for understanding visualization success, including characteristics of the person using the visualization, and their task[17].

### 2.2. Feedback intervention

**Figure 1:**
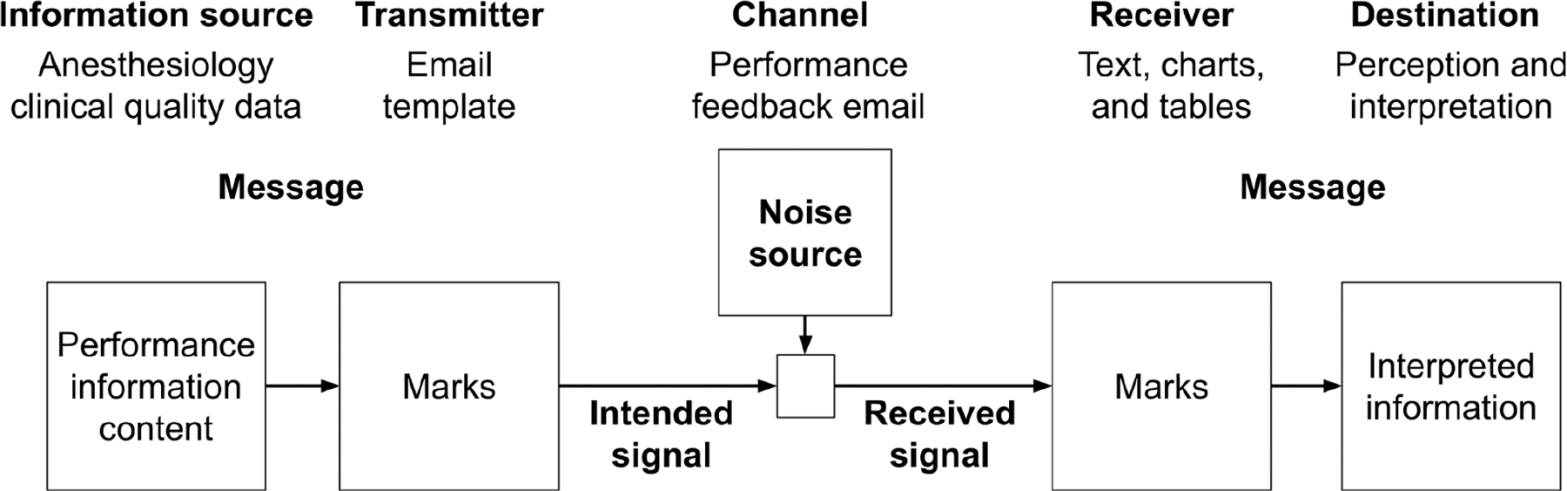
Performance communication context with email feedback intervention example

The processes through which feedback is delivered as an intervention is an important area of research inquiry. Feedback to healthcare professionals and teams can be foundationally understood as a communication process. A communication model[21] offers the constructs of source, transmitter, channel, receiver, many of which have been used in feedback theory[9,22] (Figure 1).

Feedback can be understood as a kind of communication that influences decisions and behavior. Two approaches for modeling feedback in this interventional context are the Behavior Change Intervention Ontology[23], and information value chain theory[24–26]. The Behavior Change Intervention Ontology (BCIO) models planned processes that deliver some content with an aim to influence human behavior as an outcome[23]. *Mechanisms of action* are the intermediate steps in a process through which the content influences behavior. In the case of feedback interventions, a performance summary may function as an information container for various kinds of content that are known to influence behavior, with each type of content potentially relating to a unique theoretical mechanism of action. BCIO is developed as an domain-specific ontology for intervention-related ontologies, using Basic Formal Ontology as their upper-level ontology[23].

Information value chain theory was developed for the evaluation of information systems in health-related contexts[24,26,27]. Like BCIO, information value chain theory models steps in a sequence of events that are necessary for the success of interventions. These steps represent interaction with a system and the cognitive processing of information received, followed by changes in decisions, behavior, and health-related outcomes. Information value chain theory has been used to understand the limitations of audit and feedback interventions, as well as other forms of decision support[24,25]. BCIO and information value chain theory offer constructs that may be useful for representing parts of the feedback delivery process, and the context of a performance summary.

### 2.3. Precision Feedback

Precision feedback aims to deliver high-value performance summaries by prioritizing performance information in short, actionable and motivating messages to healthcare professionals and teams[28]. For example, a message may alert a feedback recipient about a significant drop in performance, or about the achievement of a goal. A precision feedback system requires formal description of not only the information content that is available to deliver to a feedback recipient, but also a range of possible messages that could be motivating and actionable, given the recipient’s performance data.

## 3. Objective

To develop an ontology of a performance summary in a clinical performance feedback intervention for the purposes of standardizing metadata.

## 4. Methods

A research team of faculty, graduate students, and staff collaborated to create the Performance Summary Display Ontology (PSDO). We chose to use Basic Formal Ontology as an upper-level ontology, and to participate in the Open Biological and Biomedical Ontology (OBO) Foundry[29] because of the potential for semantic interoperability with BCIO and classes from other related ontologies using BFO.

We developed PSDO iteratively by 1) identifying terms for classes from behavior change theories relating to feedback interventions and cognitive theories of visualization, 2) searching for relevant existing ontologies and classes, and 3) using the terms to specify information content and visual displays in published examples of dashboard displays and feedback reports. We repeated these steps as our specifications revealed issues for refinement of terms, classes, and properties, and the need to identify additional terms.

Toward the goal of supporting a use case for precision feedback, as we refined the ontology, we developed a knowledge base and a prototype feedback system that used classes from the ontology, which enabled us to differentiate information content elements in performance data from perceived information in a feedback message.

In an additional step to assess the feasibility of PSDO to support audit and feedback research broadly, we used its terms and definitions to specify the content of published feedback reports and dashboard displays in studies of audit and feedback from a range of clinical settings[13].

## 5. Results

PSDO is a lightweight application ontology that specifies performance information content and its representations for the purpose of feedback intervention research and evaluation. PSDO contains 3 primary domains: 1) Performance information content, based on constructs from behavior change theories, 2) Marks and their qualities, based on constructs from visualization theories, and 3) roles that link marks, information content, and other emergent characteristics, as interpreted information.

Constructs from behavior change theory are based on Feedback Intervention Theory’s construct of a feedback-standard gap[30], which represents the discrepancy between current performance and a comparator that is a shared focus of Control Theory[31] and Goal-Setting Theory[32]. Constructs from visualization theory build on Zhang and Norman’s representational analysis of charts and other visual displays[19,20], and Munzner’s framework for analysis of visualizations[18,33] to describe information visualization artifacts and cognitive tasks of the viewer.

Regarding interpreted information, PSDO models the theory of ‘distributed representation’ in displays that contain two types of representational components: 1) ‘internal’ or cognitive, and 2) ‘external’ or physical-graphical entities. By representing ‘internal’ and ‘external’ entities separately we can study the alignment-of and differences-between sets of features in visualizations of performance, in order to make and test hypotheses about viewer cognition and intervention success. By making these characteristics computable PSDO enables the use of theories such as ‘information match’ between displays and the cognitive tasks they support in order to test theories of behavior change against actual changes in both cognitive processing and clinical performance.

PSDO is designed according to the principles of The Open Biological and Biomedical Ontology (OBO) Foundry[29]. We developed PSDO publicly under an open source software license and deposited the ontology in BioPortal[34]. Formal definitions for selected classes of PSDO are provided in Table 2.

### 5.1. Communication context

PSDO models information content and its representations of performance summaries in the context of clinical performance communication (Figure 1). The information source is represented as performance information content that is further represented by marks, as both transmitter and receiver, sent via some channel to a destination. The destination is interpreted information of the feedback recipient. Thus, performance information content is carried by marks and interpreted by feedback recipients.

### 5.2. Performance information content

Performance summaries must necessarily relate *performance information* to a feedback *recipient* for a specified *time interval*. Performance information contains *performance levels* (i.e. high, low, 83%, 22), that are the output of a *performance measure*. Thus, there are four necessary variables that a performance summary must relate in some way: A recipient, time interval, performance level, and performance measure.

Depending on the design of the performance summary, it may compare the recipient’s performance level to that of a peer-based comparator or an organizational goal. The potential to have multiple entities with attributed performance levels (e.g. recipient and peer group) creates the need to generalize the recipient entity to a dimension of entities with performance ascribed to them. We name this dimension *ascribees*, including the recipient and any type of comparator (e.g. goal, benchmark).

Performance summaries may contain performance levels from multiple time intervals, with multiple comparators. When these features are interpreted as information, higher-level features may be perceived to represent events such as performance trends, goal achievement, and the gain or loss of performance status among peers.

### 5.3. Marks

Following Munzner, we informally define a ‘mark’ as a graphical element that is part of a performance summary display. This can include: a single bar, a line, an axis or a cell value in a table. Further, we define a dimension as a multiset of individual marks in a performance summary display. By defining graphical elements at three levels of granularity (1) marks, (2) interpreted information, (3) and the entire performance summary display (the multiset of interpreted information) we can utilize theoretical perspectives from multiple disciplines to inform our design choices. More importantly, using this tiered understanding of graphical construction we can meaningfully describe interaction between elements in any tier, or elements across tiers.

Munzner describes marks and their ‘channels’, or qualities that control aspects of the mark’s physical manifestation, such as color[18]. Based on Munzner’s definition of a ‘channel’ as ‘a way to control the appearance of marks, independent of the dimensionality of the geometric primitive’ we hypothesize that any visual aspect of a performance summary display that can be perceived as contiguous is important to consider when designing, testing, or implementing feedback interventions. By defining performance summary elements at this level of granularity, we gain the ability to map differences between individual marks and their interpreted information to potential theoretical mechanisms of feedback interventions. For example, Feedback Intervention Theory (incorporating constructs from Control Theory and Goal-Setting Theory) asserts that a comparison between a recipient’s performance level and that of a goal or peer benchmark influences the feedback recipient’s motivation. Using Munzner’s theory of marks and channels we can informally define this comparison or ‘gap’ as a perceived distance between two marks.

### 5.4. Interpreted information

Above the level of marks, we adopt the theoretical framework of Zhang and Norman[20] to represent interpreted information that are collections of marks. Using this approach, we gain the ability to describe theoretical mechanisms in association with emergent properties, both cognitive and physical, that govern our cognitive and potentially reaction to different visualizations. Perhaps most importantly this allows us to map particular multisets of marks to cognitive tasks. For example, the achievement of a goal (represented by a recipient’s performance level improving to reach the level of the goal) is an emergent property of multiple dimensions. Cognitive analysis of goal achievement depends on properties of the lower level: marks.

To our knowledge, none of the OBO Foundry ontologies represent visual information artifacts in a way that accounts for distributed representation. PSDO represents the physical graphical components (‘marks’), the informational entities, and what those marks may come to represent to the viewer through processes of human perception, cognition or design.

### 5.5. Use Case: Precision Feedback

**Figure 3:**
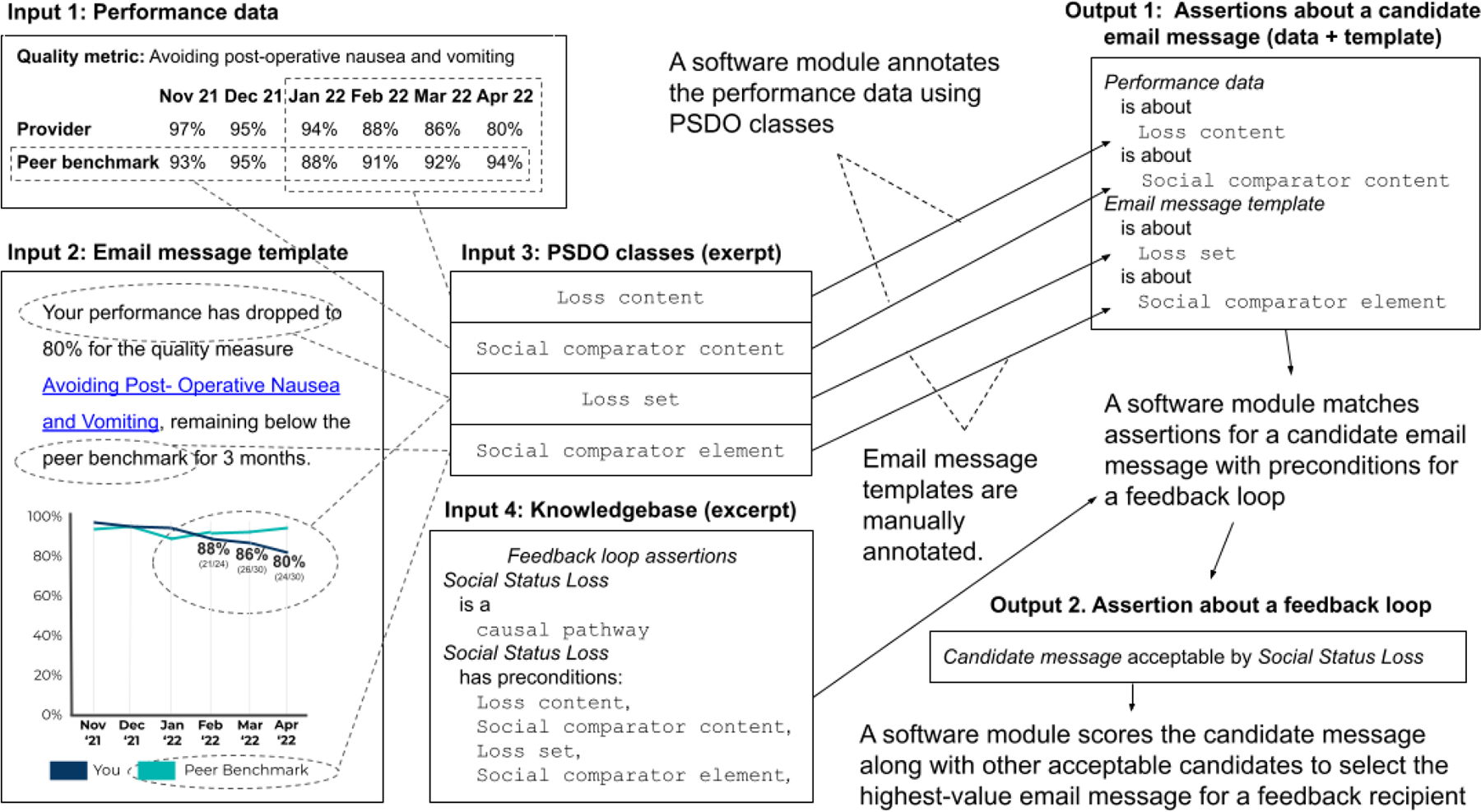
Use case for the Performance Summary Display Ontology (PSDO) by a precision feedback system

We developed a prototype precision feedback system that operates as a software pipeline with performance data as a primary input. The system uses a knowledge base composed of knowledge about feedback loops (represented as causal pathway models) with the preconditions for their availability expressed as basic characteristics of performance information content. The system first processes performance data to identify and annotate these characteristics, such as a loss of social status, or the achievement of a goal. Next, candidate email messages are created from email message templates that can be populated with the recipient’s performance data. The system assesses each candidate message to determine if it satisfies the preconditions of each feedback loop, given the performance data for the recipient. Finally, candidate messages that have one or more feedback loops available are scored to select the highest-value message (Figure 3).

## 6. Discussion

PSDO enables standardization of metadata for the study of feedback interventions in healthcare organizations and quality improvement networks. PSDO has high potential to support research and evaluation for these widely used interventions, especially for those delivered digitally via email and web-based dashboards that use visualizations to highlight important care quality gaps[5,6].

A key problem for the modeling of feedback intervention elements is the inadequate granularity of description of graphical displays of clinical performance information. Visualization studies of clinical feedback interventions typically describe visual displays at the level of a whole visual display, such as bar and line charts[35,36]. While display-level description may be adequate for the evaluation of *one size fits most* feedback interventions, it is insufficient for understanding relationships between information content, the visual elements that can strongly influence their successful interpretation[17,19], and the mechanisms of feedback interventions that are critical for their success.

Based on PSDO, performance summary information can be understood to necessarily contain data about *performance levels* related to a *feedback recipient* for one or more *time intervals* and *performance measures*[13]. Comparators can be modeled as an optional kind of *ascribee* within a performance summary that, when included in a visual display, share a dimension with the feedback recipient. Describing feedback intervention elements without this level of granularity may limit our ability to learn about the success of feedback interventions.

By using Basic Formal Ontology (BFO) as its upper-level ontology, PSDO allows for semantic interoperability with a wide net of formal scientific ontologies and serves as an easily extendable framework for future work on representation of feedback intervention elements and information visualization artifacts.

In developing PSDO, we chose to use the label *content* for terms about performance data and information, and the label *element* (and *set* where there are multiple elements) for terms about the interpreted information that recipients perceive. For example, PSDO has the terms ‘performance gap content’ and ‘performance gap set’(Table 2). These performance terms are analogous but necessary to differentiate so that a precision feedback system can reason about alternate messages with alternate kinds of interpreted information, based on the same performance data.

A primary limitation of PSDO is that it has yet to undergo a formal evaluation. We plan to initiate evaluation of PSDO in multiple phases using an ontology life cycle model[37] which recognizes differences in requirements for multiple purposes of ontology use. Our development of the precision feedback system that uses PSDO provides a primary use case with requirements that the ontology has satisfied for our prototype system.

We developed PSDO as an application ontology without the close involvement of the audit and feedback research community, which may present challenges for its broader uptake. PSDO does not yet contain important terms for metadata about performance summaries, such as the author (source) and date of delivery, as its scope has been limited to performance information, its delivery process, and information that is interpreted by recipients.

## 7. Conclusions

PSDO is an ontology for the content and delivery of performance summaries in feedback interventions. It defines performance information content, visual display elements, and interpreted information for recipients of clinical performance feedback. PSDO may enable standardization of metadata for the study of feedback interventions.

## Figures and Tables

**Figure 2:**
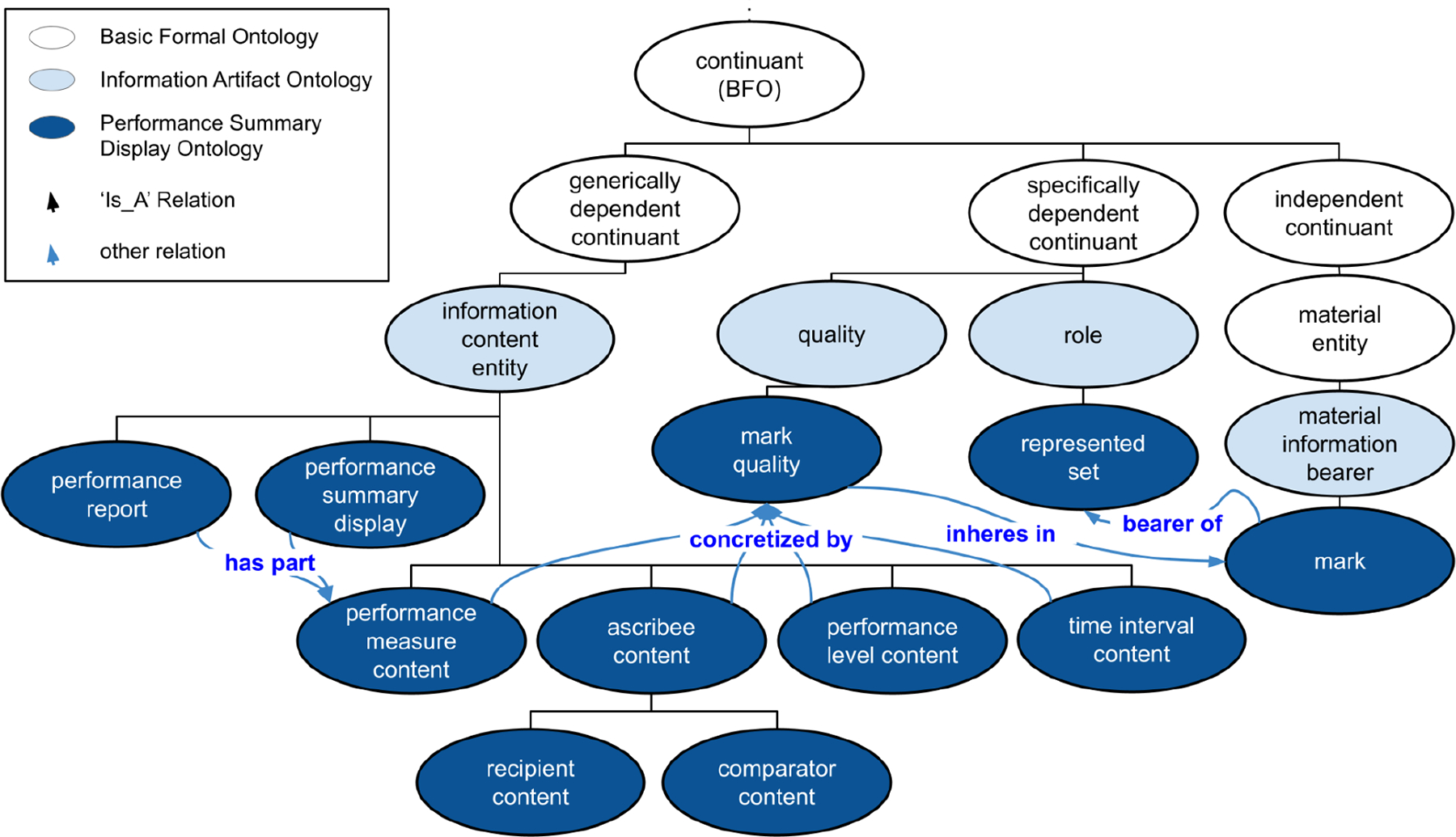
Representation of feedback intervention elements using Performance Summary Display Ontology classes

**Table 1:** Terms for the content of feedback from selected A&F frameworks

Content type	Term
Information delivered[7]	*Processes of care*
	*Patient outcomes*
	*Performance of individual provider*
	*Performance of provider group*
	*Individual patient cases*
	*Aggregate of patient cases*
	*Specific behavior to be changed*
	*Comparison provided* * Others previous performance* * Standardized guideline* * Own previous performance*
	*Graph presented*
Feedback content[12]	*Correct solution information*
	*Attainment level*
	*Velocity*
	*Normative information*
	*Goal setting type* * Difficult and specific* * Do your best*
Feedback display variables[9]	*Benchmarking*
	*Framing*
	*Graphical elements*
	*Number of metrics*
	*Patient lists*
	*Performance level*
	*Prioritization*
	*Specificity*
	*Target*
	*Timeliness*
	*Trend*
	*Usability*
	*Detailed patient-level information*
	*Qualitative data*
Information content[13]	*Measures*
	*Ascribees*
	*Performance levels*
	*Time intervals*

**Table 2:** Selected formal definitions from PSDO

Term	Definition
Achievement content	An information content entity that is about a change from a negative performance gap to a positive performance gap.
Achievement set	A represented set where the mark attributes are about a change from a negative performance gap to a positive performance gap.
Ascribee content	An information content entity that is about an entity that is ascribed a performance value.
Ascribee set	A represented set where the mark attributes are about entities that have attributed performance data.
Comparator content	Ascribee content that is used to identify a discrepancy with the performance level of the recipient of an intervention.
Comparative element	A role inhering in a mark that is disjoint with a focal element and that has the same scale type as the focal element.
Goal comparator content	Comparator content that has been ascribed a desired future performance level.
Information content entity	A generically dependent continuant that is about some thing.
Loss content	An information content entity that is about a change from a positive performance gap to a negative performance gap.
Loss set	A represented set where the mark attributes are about a change from a positive performance gap to a negative performance gap.
Mark	A material information bearer that is a basic visual element of a relational information display.
Performance content	An information content entity that is an aggregate of performance level content entities.
Performance gap content	An information content entity that is about a discrepancy between performance levels.
Performance gap set	A represented set where the mark attributes are about a discrepancy between performance levels.
Performance level content	An information content entity that is about the output value of a method of measuring performance.
Performance measure content	An information content entity about a method of measuring performance.
Performance summary display	A relational information display whose display components bear some combination of sets (roles): ascribee set, performance measure set, performance set, or time set.
Performance trend content	An information content entity that is about a change in performance
Role	A realizable entity whose manifestation brings about some result or end.
Social comparator content	Comparator content about living systems that are ascribed a performance level.
Time interval content	An information content entity that is about a unit of time.
